# Regional Differences in the Clinical Presentation of Registry-Recorded Renal Cell Carcinoma and Area-Level Healthcare Resource Indicators: A Multicenter Study in Miyazaki, Japan

**DOI:** 10.3390/healthcare14182940

**Published:** 2026-09-10

**Authors:** Shoichi Kimura, Naoki Terada, Michikazu Nakai, Toyoharu Nagata, Toshio Kamimura, Toyoharu Kamibeppu, Hiromasa Tsukino, Hironobu Wakeda, Katsuhisa Mori, Takeshi Yamasaki, Masafumi Nagano, Soichiro Fukuda, Yasuhiro Yamashita, Toshiyuki Takehara, Kentaro Kuroiwa, Takahiro Shitamura, Chie Onizuka, Atsuro Sawada, Toshiyuki Kamoto

**Affiliations:** 1 Department of Urology, Faculty of Medicine, University of Miyazaki, Miyazaki 889-1692, Japan; shoichi_kimura@med.miyazaki-u.ac.jp (S.K.);; 2 Department of Urology, Faculty of Medicine, University of Fukui, Fukui 910-1193, Japan; 3 Department of Statistics and Data Management, Faculty of Medicine, University of Miyazaki, Miyazaki 889-1692, Japan; 4 Department of Urology, Kushima City Hospital, Kushima 888-0001, Japan; 5 Department of Urology, Miyazaki Prefectural Nichinan Hospital, Nichinan 887-0013, Japan; 6 Department of Urology, Koga General Hospital, Miyazaki 880-0041, Japan; 7 Department of Urology, Junwakai Memorial Hospital, Miyazaki 880-2112, Japan; 8 Department of Urology, Chiyoda Hospital, Hyuga 883-0064, Japan; 9 Department of Urology, Kobayashi City Hospital, Kobayashi 886-8503, Japan; 10 Department of Urology, Miyakonojo Medical Center, Miyakonojo 885-0014, Japan; 11 Department of Urology, Fujimoto General Hospital, Miyakonojo 885-0055, Japan; 12 Department of Urology, Fukuda Urological Clinic, Miyazaki 880-0841, Japan; 13 Department of Urology, Miyazaki Prefectural Nobeoka Hospital, Nobeoka 882-0835, Japan; 14 Department of Urology, Ogawa Clinic, Nobeoka 882-0803, Japan; 15 Department of Urology, Miyazaki Prefectural Miyazaki Hospital, Miyazaki 880-8510, Japan; 16 Department of Urology, Saiseikai Hyuga Hospital, Kadogawa 889-0692, Japan

**Keywords:** renal cell carcinoma, registry, regional variation, healthcare resources, specialist workforce, clinical presentation

## Abstract

**Highlights:**

**What are the main findings?**

- Among 540 protocol-eligible MUCD cases, patients registered in the Northern area more often presented with predefined local symptoms and had larger tumors after adjustment for age and sex and accounting for clustering by facility.
- Clinical M1 disease was numerically more frequent in the Northern area, although the evidence for a regional difference was weaker after accounting for facility clustering and multiple comparisons.

**What are the implications of the main findings?**

- Urologist and physician availability was lower in the Northern area, but these area-level indicators do not directly measure individual healthcare access.
- Future studies should incorporate residential location, registry coverage, imaging and health-check utilization, referral pathways, and diagnostic intervals.

**Abstract:**

**Background/Objectives:** Healthcare resources are unevenly distributed across Miyazaki Prefecture, Japan. We examined regional differences in the clinical presentation of renal cell carcinoma (RCC) recorded in the Miyazaki Urological Cancer Database (MUCD), together with area-level healthcare resource indicators. **Methods:** We performed a retrospective descriptive observational analysis of prospectively collected data from the multicenter MUCD registry. After deduplication and rechecking eligibility, 540 patients with a documented diagnosis date within the study period (1 April 2019–31 March 2024) were included (Central/Southern, *n* = 333; Western, *n* = 116; Northern, *n* = 91). The three main clinical outcomes were adjusted for age and sex, with clustering by registering facility taken into account. **Results:** Presentation with predefined local symptoms occurred in 18.6%, 19.0%, and 34.1% of patients in the Central/Southern, Western, and Northern areas, respectively. Compared with Central/Southern, the adjusted odds ratio for predefined local symptoms in the Northern area was 2.30 (95% CI, 1.39–3.81). Median tumor size was 3.8, 4.4, and 5.0 cm, respectively; the adjusted mean difference for Northern versus Central/Southern was 1.24 cm (95% CI, 0.65–1.82). Clinical M1 disease was recorded in 10.2%, 12.1%, and 19.8%, respectively; the global test accounting for facility clustering yielded *p* = 0.076, and the Holm-adjusted *p* value for Northern versus Central/Southern was 0.082. Urologist density in 2024 was 8.7, 8.0, and 5.3 per 100,000 population, respectively. **Conclusions:** Regional differences were observed in the clinical presentation of MUCD-recorded RCC and in area-level healthcare resource availability. These findings are descriptive and hypothesis-generating and do not show that differences in resource availability caused the observed clinical patterns.

## 1. Introduction

In 2022, an estimated 434,000 new cases of kidney cancer were diagnosed, and 155,000 kidney cancer-related deaths occurred worldwide [1]. RCC is the most common type of kidney cancer in adults, and prognosis is strongly related to stage at diagnosis [2,3]. Many RCCs are now detected incidentally on abdominal imaging, whereas the classic triad of hematuria, flank pain, and a palpable mass is uncommon [3]. In Japan, national cancer statistics recorded 21,347 kidney cancers, excluding renal pelvis cancer, in 2019 [4]. Japanese screening studies have also shown that abdominal ultrasonography can detect otherwise asymptomatic renal tumors [5,6].

Geographic and rural–urban disparities in cancer care have been increasingly recognized, although much of the evidence comes from healthcare systems outside Japan and from cancers other than RCC [7]. A recent U.S. study of kidney cancer found modest differences in presentation and outcomes between rural and urban patients, but the underlying mechanisms were not established [8]. In Japan, national cancer statistics describe disease burden, and recent workforce research has continued to document difficulties in maintaining physician supply in rural areas [4,9]. However, few studies have directly compared RCC presentation and specialist-resource availability within a single Japanese prefecture.

Miyazaki Prefecture is geographically diverse, with secondary medical care areas that differ in population density and healthcare resources [10]. The MUCD has previously been used for multicenter urological cancer research in Miyazaki Prefecture [11]. We used physician and urologist densities, land area per urologist, and population per urologist as area-level indicators of healthcare resources rather than direct measures of individual access. Because RCC may be detected on imaging requested outside urology, urologist density was considered mainly an indicator of specialist evaluation and treatment capacity. We compared the clinical presentation of MUCD-recorded RCC cases and these resource indicators across areas defined by the location of the registering urological facility.

## 2. Materials and Methods

### 2.1. Data Source and Study Population

The MUCD is a prospective multicenter registry of newly diagnosed urological malignancies in Miyazaki Prefecture, Japan. Data were collected prospectively at 17 participating urological facilities from 1 April 2019 through 31 March 2024. The present study was a retrospective descriptive observational analysis of the prospectively collected RCC data. The protocol specified consecutive registration of eligible patients. All 17 facilities participated throughout the five-year period, and 13 contributed at least one eligible RCC case to this analysis. Registry completeness for all RCC diagnoses in Miyazaki Prefecture was not independently audited, and cases diagnosed outside the participating network were not systematically captured. The investigators had access to the complete RCC dataset exported from the MUCD for this study.

Eligible patients were adults aged ≥20 years whose first RCC diagnosis occurred during the registration period. The protocol defined the RCC diagnosis date as the date of the imaging examination establishing the diagnosis. Patients diagnosed and treated for RCC before the registration period were ineligible. The analytical unit was the patient. Bilateral, multifocal, and hereditary RCC were not separate exclusion criteria.

A total of 598 RCC records were retrieved. Potential duplicates were identified by manually cross-checking demographic and clinical information, including date of birth, sex, and referral history. When records from different participating facilities appeared to represent the same patient, the facilities were contacted when necessary, and confirmed duplicate information was reconciled and merged. The reason for each duplicate registration, including whether it resulted from inter-facility referral, was not systematically retained. After 34 duplicate registrations were merged, 564 unique patient records remained. We then rechecked all 564 records against the study diagnosis window. Fifteen had a documented diagnosis date before 1 April 2019, three after 31 March 2024, and six had no documented diagnosis date. These 24 records were excluded, leaving 540 patients in the primary cohort. No a priori sample-size calculation was performed because all eligible available cases were included.

### 2.2. Regional Classification

**Figure 1:**
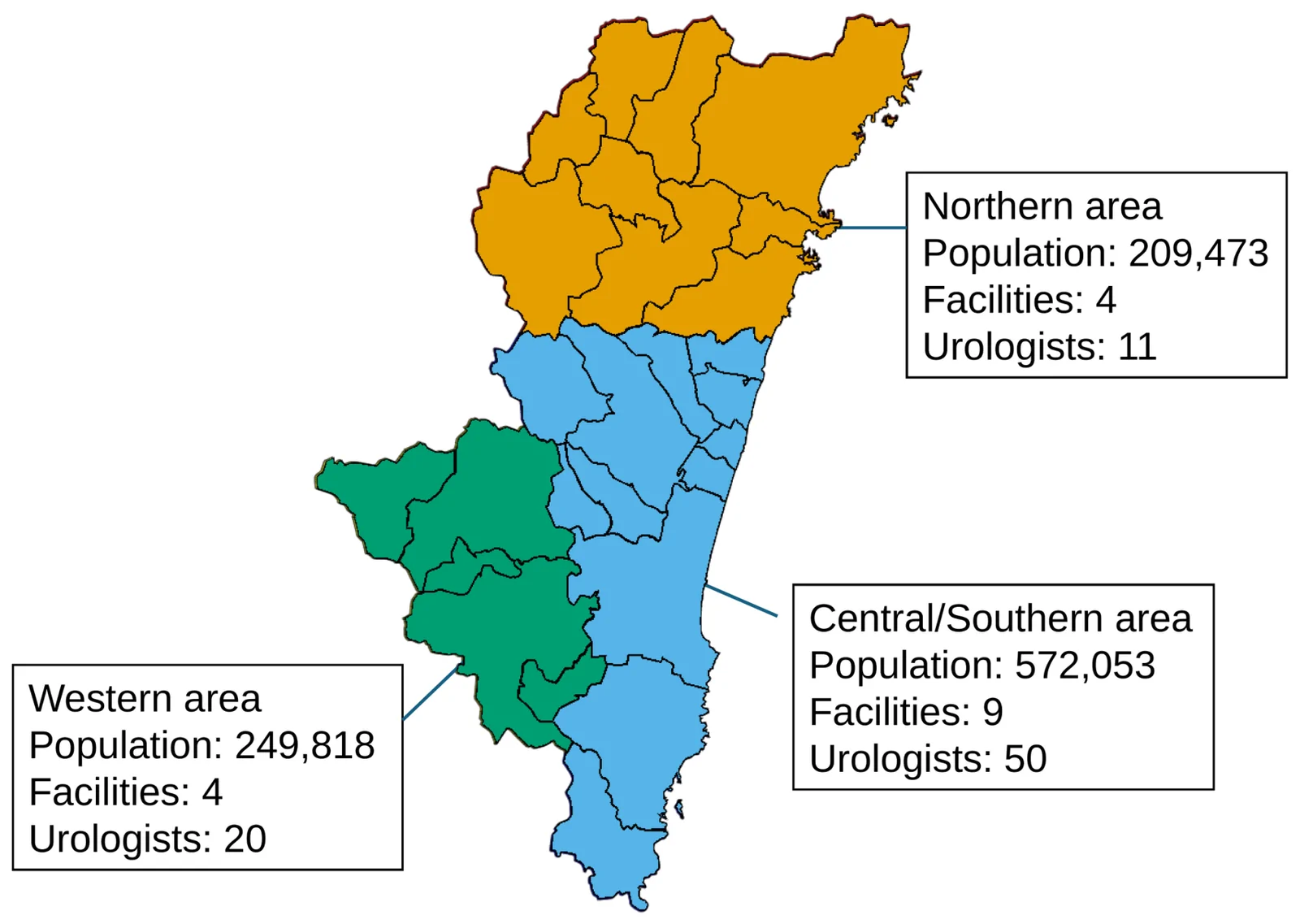
Three study regions in Miyazaki Prefecture and selected area-level healthcare resource indicators. The regions were defined from the seven secondary medical care areas. Population and urologist counts are 2024 values; facility counts refer to MUCD-participating sites. Base map: n.freemap.jp (commercial use permitted). Regional colors and annotations were added by the authors using a color-blind-safe palette.

Miyazaki Prefecture is divided into seven secondary medical care areas for regional healthcare planning [10]. For the primary analysis, these were consolidated into three study regions (Figure 1): Central/Southern (Miyazaki-Higashimorokata, Saito-Koyu, and Nichinan-Kushima), Western (Miyakonojo-Kitamorokata and Nishimorokata), and Northern (Nobeoka-Nishiusuki and Hyuga-Irigo). Central and Southern areas were grouped because urological oncology services in the Southern area are frequently provided through referral to hospitals in the Central area. Patients were assigned according to the location of the registering urological facility because residential location was not collected in the analytic dataset. Supplementary Table S2 shows the distribution of eligible cases, case-contributing facilities, and urologists across the seven original secondary medical care areas. To examine the effect of combining Central and Southern, a descriptive sensitivity analysis separating these areas was performed (Supplementary Table S3).

### 2.3. Data Collection and Healthcare Resources

Clinical variables included age, sex, presentation, ECOG performance status (PS), tumor size, clinical T/N/M category, pathological confirmation, and initial treatment. Clinical T/N/M categories were assigned at each participating institution according to the contemporaneous TNM classification (8th edition during the study period). Tumor size was the maximum diameter recorded from routine diagnostic imaging, and clinical nodal and metastatic status were assessed from routine imaging at each institution. No uniform study-specific imaging protocol or central radiologic review was used. ECOG PS was the baseline value recorded in the MUCD. Pathological confirmation was defined as a diagnostic biopsy and/or postoperative histological diagnosis recorded in the MUCD. In patients managed without biopsy or surgery, RCC could be diagnosed clinically by the treating physician on the basis of typical findings on routine computed tomography (CT) or magnetic resonance imaging (MRI).

Presentation was reconstructed from the structured registry fields and classified hierarchically into four mutually exclusive categories: (1) predefined local symptoms, when gross hematuria, abdominal/flank pain, or a palpable abdominal mass was recorded; (2) other symptoms, when another symptom was recorded without a predefined local symptom; (3) incidental/asymptomatic detection, when ultrasonography or another examination was recorded as the detection context without recorded symptoms; and (4) unknown/unclassified, when the available fields did not establish a presentation category. For the primary binary analysis, predefined local symptoms were compared with known non-local presentations; unknown/unclassified cases were excluded.

The MUCD recorded the first treatment initiated after diagnosis, but no fixed post-diagnosis time window was specified. Treatment information from structured and free-text fields was grouped as surgery, systemic therapy, radiation therapy, observation/active surveillance, or other/unclassified. More than one treatment component could be recorded for a patient, so these categories were not mutually exclusive. We also summarized whether no prespecified component, one component, or two or more components were recorded.

Regional land area and 2024 demographic characteristics were obtained from official Miyazaki Prefecture statistics [10,12]. Numbers of physicians and urologists were obtained from the 2024 Statistics of Physicians, Dentists and Pharmacists [13]. Urologists were defined using the official primary workplace and primary clinical specialty classifications. We calculated physician and urologist densities per 100,000 population, land area per urologist, and population per urologist. These measures were treated as area-level resource indicators rather than direct measures of individual healthcare access.

Because patients were classified by the location of the registering facility and residential location was unavailable, population-based regional RCC incidence could not be estimated. We therefore calculated descriptive annualized rates of eligible MUCD-registered RCC cases based on facility location. Population person-time was approximated by summing official municipal population estimates as of 1 October in 2019, 2020, 2021, 2022, and 2023, corresponding to the five registration years [12], and aggregating them to the study areas. These are registration rates, not population incidence rates.

### 2.4. Statistical Analysis

Continuous variables are presented as medians with interquartile ranges (IQRs) and were compared across the three primary regions using the Kruskal–Wallis test. Categorical variables were compared using Pearson’s chi-square test or, for sparse tables, the Fisher–Freeman–Halton exact test as appropriate. Missing or unknown values are reported explicitly and were excluded from analyses requiring a defined value; no imputation was performed.

Adjusted analyses were performed for three main clinical outcomes: presentation with predefined local symptoms, tumor size, and clinical M1 disease. Logistic regression was used for predefined local symptoms and M1 disease, and linear regression for tumor size. Each model included study region, age at diagnosis as a continuous variable, and sex. A one-way cluster-robust sandwich covariance estimator was used at the registering-facility level (13 clusters). Finite-cluster inference used t and F distributions with 12 degrees of freedom (G-1), providing a degrees-of-freedom correction for the limited number of clusters. We first tested the overall association with region and then performed pairwise regional comparisons with Holm adjustment. ECOG PS was not included because it may reflect disease severity at presentation and had missing values.

Annualized MUCD registration rates are reported per 100,000 person-years with exact Poisson 95% confidence intervals (CIs), together with rate ratios relative to the Central/Southern region. Because the numerator was based on facility location and the denominator on resident population, these rates were not treated as population incidence or used to test associations with healthcare resources.

Reporting followed the STROBE statement and its RECORD extension for studies using routinely collected health data [14,15].

ChatGPT (OpenAI, version 5.6) was used to cross-check statistical analyses performed by the authors.

## 3. Results

Of 598 retrieved RCC records, 34 duplicate registrations were reconciled and merged, leaving 564 unique patients. Applying the prespecified diagnosis window excluded 15 records with diagnosis dates before 1 April 2019, three with diagnosis dates after 31 March 2024, and six with no documented diagnosis date. The revised primary cohort therefore comprised 540 patients: 333 in the Central/Southern region, 116 in the Western region, and 91 in the Northern region. Thirteen of the 17 participating MUCD facilities contributed at least one eligible RCC case; four participating facilities had no eligible RCC cases in the analytic dataset. The distribution across the seven original secondary medical care areas is shown in Supplementary Table S2.

Clinical characteristics are summarized in Table 1. Median age was 67 years in the Central/Southern region, 72 years in the Western region, and 71 years in the Northern region. The four-category presentation distribution differed across regions (*p* < 0.001). Predefined local symptoms were recorded in 62 of 333 (18.6%) Central/Southern cases, 22 of 116 (19.0%) Western cases, and 31 of 91 (34.1%) Northern cases. Incidental/asymptomatic detection accounted for 233 (70.0%), 85 (73.3%), and 42 (46.2%) cases, respectively. Ten Central/Southern cases were unclassified for presentation; no presentation-unknown cases occurred in the other two regions.

ECOG PS was available for 466 of 540 patients (86.3%). Among patients with available ECOG PS, PS 0 accounted for 74.4% in the Central/Southern area, 81.9% in the Western area, and 62.5% in the Northern area; the multi-category distribution differed across regions (exact *p* < 0.001). Pathological confirmation was documented in 388 patients (71.9%) overall: 240/333 (72.1%) in the Central/Southern area, 94/116 (81.0%) in the Western area, and 54/91 (59.3%) in the Northern area (*p* = 0.003). Pathological confirmation was not documented in the remaining 152 patients (28.1%): 93/333 (27.9%) in the Central/Southern area, 22/116 (19.0%) in the Western area, and 37/91 (40.7%) in the Northern area.

Median tumor size was 3.8 cm [IQR 2.3–5.6] in the Central/Southern area, 4.4 cm [3.0–7.0] in the Western area, and 5.0 cm [3.4–7.1] in the Northern area (*p* < 0.001). Clinical M1 disease was recorded in 34/333 (10.2%), 14/116 (12.1%), and 18/91 (19.8%) patients, respectively (unadjusted *p* = 0.047). Adjusted comparisons are shown below.

Adjusted analyses are shown in Table 2. Compared with Central/Southern, Northern had higher odds of predefined local symptoms (OR 2.30, 95% CI, 1.39–3.81; Holm-adjusted *p* = 0.007) and a larger mean tumor size (difference 1.24 cm, 95% CI, 0.65–1.82; Holm-adjusted *p* = 0.001). For M1 disease, the Northern versus Central/Southern OR was 2.10 (95% CI, 1.10–4.01), but the global test for region yielded *p* = 0.076, and the Holm-adjusted *p* value was 0.082. The remaining pairwise comparisons are shown in Table 2.

The demographic profiles, healthcare resources, and annualized MUCD registration rates of the three study areas are summarized in Table 3. Urologist density in 2024 was 8.7 per 100,000 population in the Central/Southern area, 8.0 in the Western area, and 5.3 in the Northern area; the corresponding physician densities were 329.7, 202.5, and 195.3 per 100,000 population. Land area per urologist was greatest in the Northern area (289.7 km^2^).

Using summed annual municipal populations for 2019–2023, the annualized rates of eligible MUCD-registered cases based on facility location were 11.37 (95% CI, 10.18–12.66), 9.14 (7.55–10.96), and 8.29 (6.68–10.18) per 100,000 person-years in the Central/Southern, Western, and Northern areas, respectively. Because residential region was unavailable, these estimates should not be interpreted as population-based RCC incidence. Among the 526 patients for whom an initial-treatment facility could be mapped to a study region, 521 (99.0%) received initial treatment in the same study region as the registering facility; five received initial treatment in a different study region. This does not resolve the absence of residential-location information.

## 4. Discussion

This multicenter registry-based study showed regional differences in RCC presentation and area-level healthcare resources in Miyazaki Prefecture. After adjustment for age and sex and accounting for facility clustering, patients registered in the Northern area more often presented with predefined local symptoms and had larger tumors than those registered in the Central/Southern area. M1 disease was numerically more frequent in the Northern area, although the evidence for this difference was weaker after facility clustering and multiple-comparison adjustment. The Northern area also had lower physician and urologist densities and a larger land area per urologist in 2024. Because individual healthcare access was not measured, these resource differences cannot be used to explain the clinical findings.

The difference in presentation was largely due to a lower proportion of incidental/asymptomatic detection in the Northern area. RCC is now commonly detected incidentally on imaging, and Japanese screening studies have shown that abdominal ultrasonography can detect asymptomatic renal tumors [3,5,6]. Incidental/asymptomatic detection accounted for 46.2% of Northern cases, compared with about 70% in the other two areas. Imaging use, health-check participation, primary-care use, and referral pathways may differ among areas, but these factors were not recorded in the MUCD.

The workforce measures in this study are area-level indicators and should not be interpreted as direct measures of healthcare access. RCC may be detected on imaging requested outside urology, so urologist density is more relevant to specialist evaluation and treatment capacity than to the first opportunity for detection. Physician density provides broader regional context but is also indirect. Recent Japanese workforce research has highlighted continuing shortages in rural areas [9]. With only three study areas, we could not test a statistical relationship between workforce supply and individual RCC presentation.

The annualized MUCD registration rate was lower in the Northern area than in the Central/Southern area when five annual population estimates were used. These values should not be interpreted as regional RCC incidence because cases were assigned by registering-facility location, whereas the denominators represent residents. Initial treatment was given within the registering study area for 99.0% of patients with mappable treatment-facility data, but referral across areas before registration could not be assessed. The observed pattern could reflect differences in registry completeness, care outside the MUCD network or outside Miyazaki Prefecture, regional misclassification, or true regional variation in RCC occurrence or risk factors.

This study has several limitations. First, residential location was unavailable, creating a numerator–denominator mismatch for regional registration rates. Second, the proportion of all Miyazaki RCC cases captured by MUCD is unknown; although the protocol specified consecutive registration and all 17 facilities participated throughout the registration period, completeness was not independently audited. Third, diagnosis, imaging, and staging were performed in routine practice at each institution without central radiologic review. Fourth, pathological confirmation was not available for all cases and differed by region. Fifth, presentation and ECOG PS had missing data, and non-random missingness cannot be excluded. Sixth, 2024 workforce counts are cross-sectional snapshots and may not represent resource availability throughout 2019–2024. Seventh, only 13 facilities contributed eligible RCC cases and there were 66 M1 events, which limited the precision of adjusted estimates, particularly for M1 disease. Finally, this study did not measure imaging utilization, health-check participation, diagnostic intervals, or referral pathways and cannot establish causal mechanisms. The findings may not be generalizable beyond Miyazaki Prefecture or to population-based RCC cohorts because MUCD is a facility-based registry.

## 5. Conclusions

This multicenter registry-based study identified regional differences in the clinical presentation of MUCD-recorded RCC in Miyazaki Prefecture. After adjustment for age and sex and accounting for facility clustering, patients registered in the Northern area more often presented with predefined local symptoms and had larger tumors than those in the Central/Southern area. M1 disease was numerically more frequent in the Northern area, although the evidence for this difference was weaker after accounting for facility clustering and adjustment for multiple comparisons. The Northern area had lower physician and urologist availability, but individual healthcare access was not measured and causality cannot be inferred. Further studies incorporating residential location, registry coverage, imaging and health-check use, referral pathways, and diagnostic intervals are needed to clarify these regional differences.

## Figures and Tables

**Table 1:** Characteristics of the 540 patients with RCC in the three study areas.

Characteristic	Central/Southern (*n* = 333)	Western (*n* = 116)	Northern (*n* = 91)	*p* Value
Age, years, median [IQR]	67 [57–75]	72 [65–77]	71 [64–77]	<0.001 ^a^
Age ≥ 65 years	188 (56.5)	89 (76.7)	65 (71.4)	<0.001 ^b^
Male sex	224 (67.3)	82 (70.7)	57 (62.6)	0.472 ^b^
Presentation category				<0.001 ^c^
Incidental/asymptomatic	233 (70.0)	85 (73.3)	42 (46.2)	
Predefined local symptoms	62 (18.6)	22 (19.0)	31 (34.1)	
Other symptoms	28 (8.4)	9 (7.8)	18 (19.8)	
Unknown/unclassified	10 (3.0)	0	0	
Pathological confirmation documented	240 (72.1)	94 (81.0)	54 (59.3)	0.003 ^b^
ECOG PS among available cases				<0.001 ^c^
Available *n*	281	105	80	
PS 0	209 (74.4)	86 (81.9)	50 (62.5)	
PS 1	50 (17.8)	10 (9.5)	22 (27.5)	
PS 2	17 (6.0)	2 (1.9)	3 (3.8)	
PS 3	3 (1.1)	7 (6.7)	2 (2.5)	
PS 4	2 (0.7)	0	3 (3.8)	
Unknown, *n* (% of full region)	52 (15.6)	11 (9.5)	11 (12.1)	
Tumor size, cm, median [IQR]	3.8 [2.3–5.6]	4.4 [3.0–7.0]	5.0 [3.4–7.1]	<0.001 ^a^
Clinical T stage				0.001 ^b^
cT1a	178 (53.5)	50 (43.1)	37 (40.7)	
cT1b	97 (29.1)	26 (22.4)	31 (34.1)	
cT2–T4	58 (17.4)	40 (34.5)	23 (25.3)	
Clinical N+, *n* (%)	18 (5.4)	12 (10.3)	8 (8.8)	0.155 ^b^
Clinical M1, *n* (%)	34 (10.2)	14 (12.1)	18 (19.8)	0.047 ^b^
Recorded initial-treatment components				
Surgery	245 (73.6)	98 (84.5)	56 (61.5)	0.001 ^b^
Systemic therapy	29 (8.7)	11 (9.5)	16 (17.6)	0.046 ^b^
Radiation therapy	4 (1.2)	2 (1.7)	1 (1.1)	0.863 ^d^
Observation/active surveillance	41 (12.3)	7 (6.0)	15 (16.5)	0.056 ^b^
Other/unclassified	5 (1.5)	0	3 (3.3)	0.154 ^d^
Number of prespecified treatment components				0.009 ^c^
None recorded	23 (6.9)	0	4 (4.4)	
One	296 (88.9)	114 (98.3)	83 (91.2)	
≥2	14 (4.2)	2 (1.7)	4 (4.4)	

Data are shown as *n* (%) or median [IQR]. ECOG PS percentages for categories 0–4 use patients with available values as the denominator; the Unknown row uses the full regional sample. Age, sex, tumor size, cT/cN/cM, and pathological confirmation were available for all 540 patients. Initial treatment categories were not mutually exclusive; “None recorded” indicates that no prespecified treatment component was recorded. ^a^ Kruskal–Wallis test; ^b^ Pearson chi-square test; ^c^ Fisher–Freeman–Halton exact test for multi-category tables; ^d^ Fisher–Freeman–Halton exact test for sparse binary treatment-component tables. Unadjusted ORs for surgery versus Central/Southern were 1.96 (95% CI, 1.12–3.42) for Western and 0.57 (95% CI, 0.35–0.94) for Northern. ECOG PS, Eastern Cooperative Oncology Group performance status; IQR, interquartile range; RCC, renal cell carcinoma.

**Table 2:** Age- and sex-adjusted comparisons of the three main clinical outcomes by study area.

Outcome	Contrast	Adjusted Estimate (95% CI); Holm-Adjusted *p*
Predefined local symptoms, OR (*n* = 530; global *p* = 0.001)	Western vs. Central/Southern	OR 1.04 (0.68–1.61); *p* = 0.834
	Northern vs. Central/Southern	OR 2.30 (1.39–3.81); *p* = 0.007
	Northern vs. Western	OR 2.20 (1.53–3.16); *p* = 0.001
Tumor size, mean difference (cm) (*n* = 540; global *p* < 0.001)	Western vs. Central/Southern	0.46 (0.27–0.65); *p* < 0.001
	Northern vs. Central/Southern	1.24 (0.65–1.82); *p* = 0.001
	Northern vs. Western	0.78 (0.27–1.29); *p* = 0.006
Clinical M1 disease, OR (*n* = 540; global *p* = 0.076)	Western vs. Central/Southern	OR 1.11 (0.53–2.35); *p* = 0.762
	Northern vs. Central/Southern	OR 2.10 (1.10–4.01); *p* = 0.082
	Northern vs. Western	OR 1.89 (0.81–4.42); *p* = 0.256

Models were adjusted for age at diagnosis (continuous) and sex. A one-way cluster-robust sandwich covariance estimator was used at the registering-facility level (13 clusters), with finite-cluster t/F inference based on 12 degrees of freedom. Logistic regression was used for predefined local symptoms and M1 disease, and linear regression for tumor size. Ten presentation-unknown cases were excluded from the local-symptom model. Pairwise *p* values were Holm-adjusted; 95% CIs were not adjusted for multiple comparisons. CI, confidence interval; OR, odds ratio.

**Table 3:** Demographics, healthcare resources, and annualized MUCD registration rates by study area.

Indicator	Central/Southern	Western	Northern
Eligible MUCD RCC cases, 1 April 2019–31 March 2024	333	116	91
Summed population person-years, 1 October 2019–2023	2,928,446	1,269,803	1,097,283
Annualized MUCD registration rate per 100,000 person-years (95% CI)	11.37 (10.18–12.66)	9.14 (7.55–10.96)	8.29 (6.68–10.18)
Registration-rate ratio vs Central/Southern (95% CI)	1.00	0.80 (0.65–0.99)	0.73 (0.58–0.92)
2024 population	572,053	249,818	209,473
2024 land area (km^2^)	2855.0	1695.1	3186.3
2024 population aged ≥65 years (%)	32.7	34.4	37.1
MUCD participating facilities (*n*)	9	4	4
2024 urologists (*n*)	50	20	11
2024 physicians in medical facilities (*n*)	1886	506	409
2024 urologist density per 100,000	8.7	8.0	5.3
2024 physician density per 100,000	329.7	202.5	195.3
2024 land area per urologist (km^2^)	57.1	84.8	289.7
2024 population per urologist	11,441	12,491	19,043

Population person-time for registration-rate calculations was approximated by summing official municipal population estimates as of 1 October in 2019, 2020, 2021, 2022, and 2023. Exact Poisson CIs are shown for rates. Rate-ratio CIs are log-scale approximations. Cases are classified by registering-facility location, not patient residence; therefore, these are descriptive MUCD registration rates and not population incidence rates. 2024 population, land area, age structure, and workforce counts are cross-sectional contextual indicators. Sources: Miyazaki Prefectural Government [10,12] and Ministry of Health, Labour and Welfare [13].

## Data Availability

The patient-level MUCD dataset is not publicly available because of privacy and ethical restrictions. The study protocol, deidentified aggregate data, and analytical code may be available from the corresponding author upon reasonable request, subject to institutional requirements.

## Supplementary Materials

The following supporting information can be downloaded at: https://www.mdpi.com/article/10.3390/healthcare14182940/s1, Table S1: Eligibility assessment and missing data; Table S2: Protocol-eligible cases across the seven secondary medical care areas; Table S3: Sensitivity analysis separating the Central and Southern regions; Table S4: Annual population denominators and MUCD registration rates. Completed STROBE and RECORD checklists are provided separately.

## Abbreviations

The following abbreviations are used in this manuscript:

CI	confidence interval
ECOG	Eastern Cooperative Oncology Group
IQR	interquartile range
MUCD	Miyazaki Urological Cancer Database
OR	odds ratio
PS	performance status
RCC	renal cell carcinoma
