# Supplementary material for: Regional Differences in the Clinical Presentation of Registry-Recorded Renal Cell Carcinoma and Area-Level Healthcare Resource Indicators: A Multicenter Study in Miyazaki, Japan
Source: Healthcare (Basel). 2026 Sep 10;14(18):2940. doi: 10.3390/healthcare14182940 (PMC13606433; doi:10.3390/healthcare14182940)
Supplement: Supplementary file 1 [file healthcare-14-02940-s001.zip › healthcare-4477715-supplementary.pdf]

Supplementary Table S1. Eligibility assessment and missing data

Item	Overall	Central/Southern	Western	Northern
Retrieved RCC records	598	—	—	—
Duplicate registrations merged	34	—	—	—
Unique records after deduplication	564	—	—	—
Diagnosis before 1 Apr 2019	15	—	—	—
Diagnosis after 31 Mar 2024	3	—	—	—
Diagnosis date missing	6	—	—	—
Primary protocol-eligible cohort	540	333	116	91
Presentation unknown/unclassified	10	10	0	0
ECOG PS unknown	74	52	11	11

Records outside the study period or without a documented diagnosis date were excluded.

Supplementary Table S2. Protocol-eligible cases across the seven secondary medical care areas

Secondary medical care area	Primary study region	Eligible RCC cases	Case-contributing facilities	2024 urologists (n)
Miyazaki-Higashimorokata	Central/Southern	315	5	42
Saito-Koyu	Central/Southern	0	0	5
Nichinan-Kushima	Central/Southern	18	2	3
Miyakonojo-Kitamorokata	Western	91	2	14
Nishimorokata	Western	25	1	6
Nobeoka-Nishiusuki	Northern	80	2	6
Hyuga-Irigo	Northern	11	1	5

Case-contributing facilities are the 13 participating sites with at least one eligible RCC case. Urologist counts are 2024 values from official statistics [13].

Supplementary Table S3. Sensitivity analysis separating the Central and Southern regions.

Region	n	Predefined local symptoms	Tumor size, median (cm)	Clinical M1
Central	315	58 (18.4%)	3.8	34 (10.8%)
Southern	18	4 (22.2%)	5.0	0 (0%)
Western	116	22 (19.0%)	4.4	14 (12.1%)
Northern	91	31 (34.1%)	5.0	18 (19.8%)

The Southern region was small (n=18); the results are descriptive.

Supplementary Table S4. Annual population denominators and MUCD registration rates.

Year (1 October)	Central/Southern	Western	Northern
2019	589,932	257,225	224,920
2020	590,432	256,178	222,966
2021	587,013	254,091	219,854
2022	583,164	252,173	216,434
2023	577,905	250,136	213,109
Summed person-years	2,928,446	1,269,803	1,097,283
Eligible MUCD cases	333	116	91
Rate/100,000 person-years (95% CI)	11.37 (10.18–12.66)	9.14 (7.55–10.96)	8.29 (6.68–10.18)
Rate ratio vs Central/Southern (95% CI)	1.00	0.80 (0.65–0.99)	0.73 (0.58–0.92)

Source: Miyazaki Prefectural Government [12]. Rates are based on registering-facility location.
